# An LC-MS Approach to Quantitative Measurement of Ammonia Isotopologues

**DOI:** 10.1038/s41598-017-09993-6

**Published:** 2017-09-04

**Authors:** Jessica B. Spinelli, Liam P. Kelley, Marcia C. Haigis

**Affiliations:** 1 Department of Cell Biology, Harvard Medical School, Boston, MA 02115 USA; 2 Ludwig Center at Harvard, Harvard Medical School, Boston, MA 02115 USA

## Abstract

Ammonia is a fundamental aspect of metabolism spanning all of phylogeny. Metabolomics, including metabolic tracing studies, are an integral part of elucidating the role of ammonia in these systems. However, current methods for measurement of ammonia are spectrophotometric, and cannot distinguish isotopologues of ammonia, significantly limiting metabolic tracing studies. Here, we describe a novel LC-MS-based method that quantitatively assesses both ^14^N-and ^15^N-isotopologues of ammonia in polar metabolite extracts. This assay (1) quantitatively measures the concentration of ammonia in polar metabolite isolates used for metabolomic studies, and (2) accurately determines the percent isotope abundance of ^15^N-ammonia in a cell lysate for ^15^N-isotope tracing studies. We apply this assay to quantitatively measure glutamine-derived ammonia in lung cancer cell lines with differential expression of glutaminase.

## Introduction

Quantitative measurement of ammonia is paramount in a broad spectrum of research topics. As the most reduced form of inorganic nitrogen in nature, ammonia plays a pivotal role in the nitrogen cycle among aquatic life, plants, animals and microorggranisms, as well as in the geochemical formation of the early Earth.^1^ In addition, ammonia is an essential nitrogen source for yeast and E. *coli*, and deprivation of ammonia impairs their growth and survival^2, 3^. Ammonia is also a normal by-product of nucleotide and amino acid metabolism, and systemic organismal levels are tightly regulated^4, 5^. Furthermore, the microbiome generates an abundance of ammonia, and contributes to >50% (~3 g/day) of the total ammonia in mammals^6^. Dysfunction of ammonia metabolism underlies many diseases including cancer, urea cycle disorders, hepatic encephalopathy, and cerebral dysfunction such as, intracranial hypertension, seizures, and ataxia^7, 8^. Thus, assays to accurately measure ammonia would deepen our understanding of ammonia in biology.

Current techniques for ammonia quantification include ion-selective electrodes (ISEs), enzyme-based assays, and colorimetric assays. However, these methods for ammonia measurement have limitations, and there is no technique available that specifically and sensitively detects ammonia. ISEs are complicated by cross-ion interferences, while enzyme-based assays may be sensitive to factors such as salt concentration and metabolites that compete with or allosterically modulate enzyme activity^9, 10^. In addition, metabolites commonplace to biology interfere with readouts in colorimetric assays through overlapping absorption spectra^11^. Therefore, with current technology, ammonia cannot be specifically measured in biological samples. Moreover, while many assays measure ammonia, there is no assay capable of distinguishing between isotopologues of ammonia (^14^NH_3_ and^15^NH_3_) for metabolic tracing studies.

Metabolic tracing experiments are commonly used to determine the biosynthetic pathways utilized by a cell to generate a metabolite^12^. In a metabolic tracing assay, cells are incubated with a stable isotopologues (usually containing ^13^C or ^15^N) of the metabolite of interest. Polar metabolites are then extracted and analyzed with liquid chromatography coupled to mass spectrometry (LC-MS). The derivatives of the labeled metabolite acquire ^13^C and/or ^15^N, and therefore are identified by a shift in mass-to-charge ratio (m/z). Such experiments have been utilized to determine TCA cycle anaplerosis, and nitrogen sources for nucleotide synthesis^13, 14^. However, unlike most polar metabolites, ammonia is not detected by LC-MS and is therefore neglected in metabolic tracing studies.

**Figure 1:**
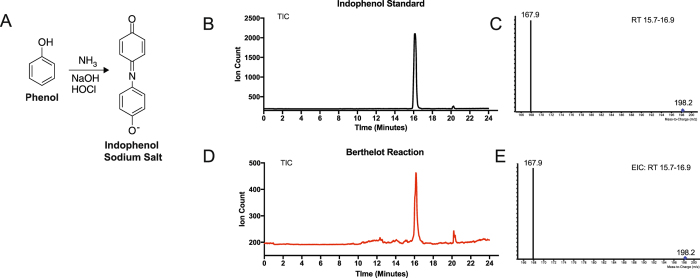
Indophenol Generated from The Berthelot Reaction is detected with LC-MS. (**A**) Schematic of the Berthelot Reaction, which generates indophenol from ammonia. (**B**) Total ion chromatogram of an indophenol standard (1*u*g/mL). (**C**) Mass spectrum for the retention time window 15.7–16.9 minutes of the total ion chromatogram of the indophenol standard (**D**) Total ion chromatogram of 500 *μ*M ammonia after the Berthelot reaction. (**E**) Mass spectrum for the retention time window 15.7–16.9 minutes of the total ion chromatogram of the Berthelot reaction.

The Berthelot reaction is a colorimetric assay utilized to measure ammonia, which was originally developed by chemist Pierre Eugene Marcellin Berthelot (1827–1907)^15^. In this reaction, an oxidative phenolic coupling generates the compound indophenol from ammonia (Fig. 1A). Indophenol is highly conjugated, and absorbs strongly between 630 and 720 nm, enabling spectrophotometric detection^16^. This reaction has been applied to measure ammonia in complex matrices, such as blood, soil, and seawater^17–19^. This assay is also the standard analysis used in hospital Blood Urea Nitrogen (BUN) exams, in which urea is converted to ammonia via Urease and subsequently converted to indophenol for detection^20^. However, similar to the limitations of other colorimetric assays, the Berthelot reaction for ammonia detection is complicated by competing molecules that interfere with the reaction or absorb on the same spectra as indophenol, such as chlorophyll^11, 21^.

We hypothesized that indophenol generated in the Berthelot reaction could be amenable to LC-MS, allowing for the direct and specific quantification of ammonia in a manner that filters out background reactions. We developed an LC-MS coupled Berthelot assay, which can be performed on polar metabolite extracts and other complex biological samples. Furthermore, we showed that the MS Berthelot assay allowed detection of isotopologues of ammonia (^14^NH_3_ and ^15^NH_3_). In this study we applied this assay to quantify the contribution of ^15^N-(amide)-glutamine to total cellular ammonia.

## Results

### The Berthelot Reaction Generates Indophenol and is Sensitively Detected by LC-MS

**Figure 2:**
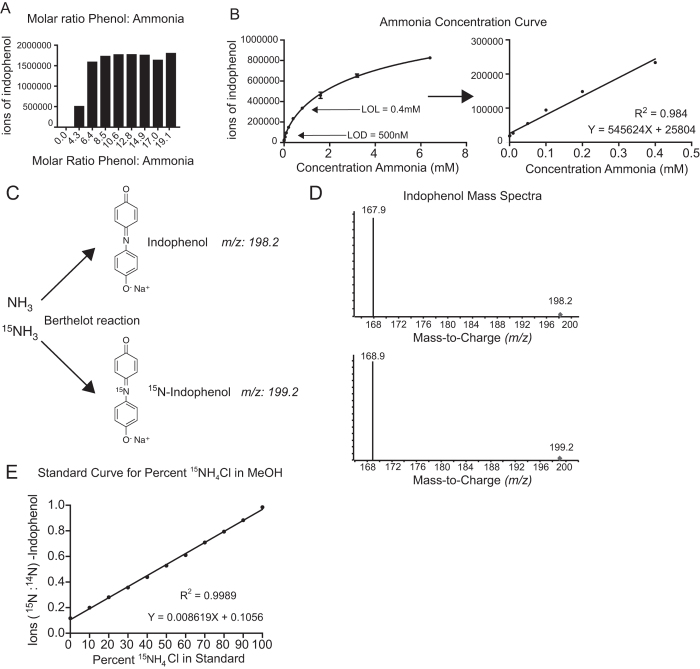
The Berthelot Reaction Paired with LC-MS Analysis Sensitively Measures Ammonia. (**A**) Optimization of the molar ratio of phenol to ammonia for maximal readout of indophenol after the Berthelot reaction. (**B**) Concentration curve of ammonia after the Berthelot reaction measured as indophenol with LC-MS. Ammonia standards were made in 80% methanol, n=3 samples per concentration. LOD: Limit of detection, LOL: limit of linearity. (**C**) Schematic of the Berthelot reaction on NH_3_ and ^15^NH_3_, which generate indophenol and ^15^N-indophenol respectively. (**D**) Mass spectra of 500 *μ*M NH_3_ (top) and 500 *μ*M ^15^NH_3_ after the Berthelot reaction in 80% methanol. (**E**) Standard curve of percent ^15^NH_3_: NH_3_ in a 500 *μ*M ammonia solution generated in 80% methanol treated with the berthelot reaction. The resulting ratio of ^15^N-indophenol: ^14^N-indophenol are plotted as a function of the expected percent ^15^N-ammonia.

We developed a mass spectrometry-based (MS) Berthelot assay to quantitatively measure ammonia. First, we assayed a pure indophenol standard using LC-MS (Fig. 1B,C). Next, we assayed products from the Berthelot reaction, which we adapted to be performed in 80:20 methanol:water, allowing for compatibility with polar metabolite extracts. We observed the same peak and MRM transition (198.1–167.1) as the pure indophenol standard (Fig. 1D,E). Next, we optimized the molar ratio of the reactant phenol to ammonia being measured (Fig. 2A). In this assay, we tested 50 mM NH_4_Cl, which is far greater than any physiological concentration observed in biology^7^. To ensure reaction completion, a minimum volumetric ratio of 4:1 for each reactant solution to ammonia was required. Therefore, to ensure all measurements are quantitative, we used a volumetric ratio of 5:1 in our assays. Next, we assessed the sensitivity of this assay to low ammonia concentrations (Fig. 2B) and determined the lower limit of ammonia detection to be 500 nM, with a limit of linearity of 0.4 mM. Therefore, this assay is optimal for quantitatively measuring ammonia concentrations in physiological ranges for human plasma (~35 μM) and in fresh water, in which ammonia concentration is very low.

### The MS Berthelot Assay Accurately Detects Ammonia Isotopologues (^14^NH_3_ and ^15^NH_3_)

We tested whether ammonia isotopologues (^14^NH_3_ and ^15^NH_3_) can be distinguished by LC-MS (Fig. 2C). We performed the Berthelot reaction on both isotopologues of ammonia and found distinct mass spectra: ^14^NH_3_ generates ^14^N-indophenol with an MRM transition of 198.2–167.9, and ^15^NH_3_ generates ^15^N-indophenol with an MRM transition of 199.2–168.9 (Fig. 2D). Next, we tested whether the assay can accurately calculate the percent abundance of ^14^N versus ^15^N-labeled ammonia (Fig. 2E). The Berthelot reaction was performed with ratios of 500 μM ^14^N- and ^15^N- ammonia mixtures. The resulting standard curve was linear, R^2^ = 0.9989, and therefore accurately measures ratios of ^14^N: ^15^N-ammonia.

### The MS Berthelot Assay Accurately Measures Ammonia in Cell Lysates

**Figure 3:**
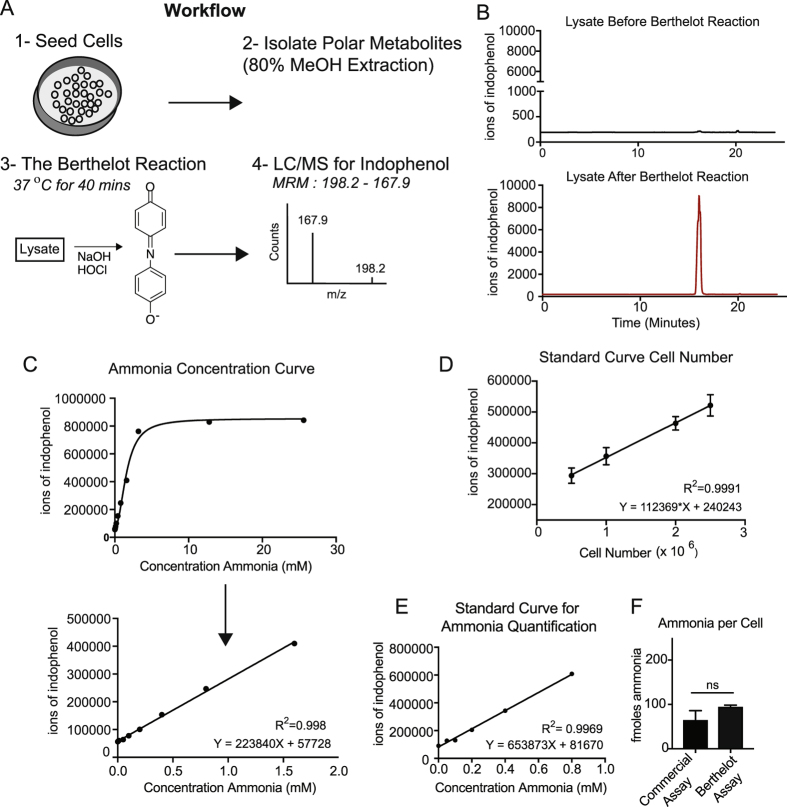
Assay development in cellular lysates. (**A**) Schematic of the work flow for measuring ammonia from a cellular lysate. (**B**) Total ion chromatogram for the MRM transition of 198.2: 167.9 in a cellular lysate extracted with 80:20 methanol:water (top) and a cellular lysate after the Berthelot reaction (bottom). (**C**) Concentration curve of the Berthelot reaction on samples with increasing ammonia spiked into cellular lysate from 3 × 10^6^ 293T cells. (**D**) Standard curve of the Berthelot reaction performed on cellular lysates from increasing cell number. (**E**) Standard curve of the Berthelot reaction performed on cellular lysates with spiked in concentrations of ammonia. (**F**) Measurement of moles of ammonia per cell using the standard addition technique with the Indophenol assay, and in parallel from the same lysate, measurement of ammonia using a standard assay (n = 3 replicates).

We tested whether the MS Berthelot assay could quantify ammonia from cell lysates, a more complex matrix comprised of many metabolites that may potentially interfere with the Berthelot reaction. Importantly, we performed the MS Berthelot assay using lysates extracted in 80% methanol, amenable for metabolite profiling (Fig. 3A). To ensure completion of the reaction in a cell lysate, we used a volumetric ratio of 10:1 of each solvent to lysate. First, we assayed the specificity of indophenol for the MS Berthelot assay. Indophenol was undetectable in mammalian cell lysates, and was only detected after the Berthelot reaction (Fig. 3D). Thus, as indophenol is not a mammalian metabolite, it can be distinctly measured after reaction with ammonia in the complex matrix of a polar metabolite extraction.

We also evaluated the sensitivity of ammonia detection from mammalian cells. We spiked a concentration curve of ammonia into 293T lysates, generating a linear standard curve with an R^2^ value of 0.998 (Fig. 3C). Furthermore, ammonia increased linearly with a dynamic cell range of 500,000 to 2.5 million cells (R^2^ = 0.9991) (Fig. 3D). To directly assay ammonia concentrations within mammalian cells, we performed the MS Berthelot assay from a cell lysate not spiked with ammonia. We used the standard addition technique to generate a standard curve by spiking ammonia into 293T cell lysates (0.0–0.8 mM ammonia) (Fig. 3E). In 293T cells, we measured 100 femtomoles of ammonia per cell (Fig. 3F), consistent with levels detected using a commercial ammonia assay.

### The MS Berthelot Assay Distinctly Measures Ammonia Isotopologues in Cell Lysates

**Figure 4:**
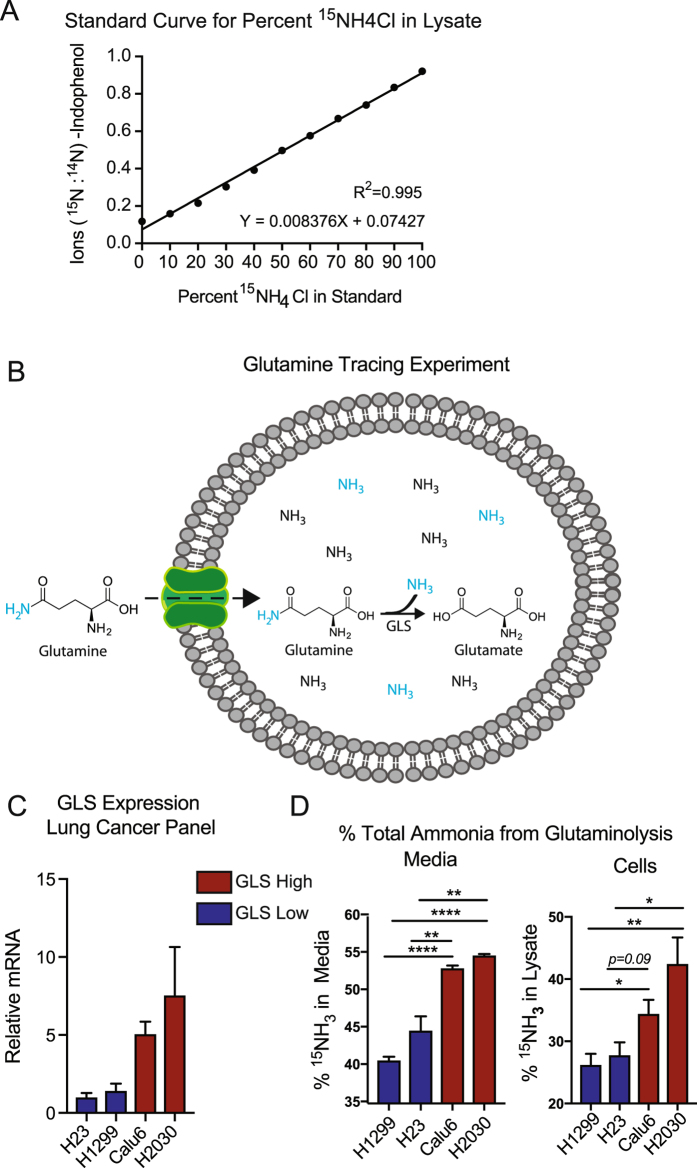
Application of Indophenol Assay to Quantitatively Measure Glutamine-derived Ammonia in Lung Cancer Cell Lines. (**A**) Standard curve of percent ^15^NH_3_: NH_3_ in a 500 *μ*M ammonia solution generated in cellular lysate from 293T cells treated with the Berthelot reaction. The resulting ratio of ^15^N-indophenol: ^14^N-indophenol are plotted as a function of the expected percent ^15^N-ammonia. (**B**) Schematic of the metabolic tracing experiment to elucidate glutamine contribution to cellular ammonia pools. Blue represents the ^15^N-isotope. (**C**) mRNA levels of glutaminase in lung cancer cell line panel. n = 3 replicates. (**D**) Percent ^15^N-ammonia after treatment with 2.0 mM ^15^N-(amide)-glutamine. Measurements were taken in the media and in cellular lysates of the lung cancer panel, n = 3 replicates per cell line.

Next, we tested whether the MS Berthelot assay accurately measured ratios of ^14^N/^15^N-ammonia spiked into a cell lysate. Indeed, with R^2^ = 0.995, this assay accurately measures the ratio of ammonia isotopologues (Fig. 4A). We next applied isotope-tracing studies to test whether ammonia as a by-product of metabolic reactions can be detected using a biologically relevant cellular system. Glutamine is commonly catabolized by the ubiquitous enzyme glutaminase (GLS) to generate glutamate and ammonia (Fig. 4B). Lung cancer cell lines vary in GLS expression, and therefore represent an excellent model to study glutamine-derived ammonia using this assay (Fig. 4C). We treated GLS high (Calu6, H2030) and GLS low (H23, H1299) lung cancer cell lines with the stable isotope ^15^N-(amide)-glutamine, which liberates ^15^N-ammonia in the process of glutaminolysis. We performed the MS Berthelot assay on media from cultured cells, as well as cell lysates. As anticipated, cell lines with high GLS expression contributed more glutamine-derived ammonia to the total ammonia pool in media (~55%) and in cells (~45%), compared to GLS low cell lines, which contributed 45% and 30% to media and cellular ammonia, respectively (Fig. 4D).

## Discussion

Here we describe the development of a novel mass spectrometry assay, MS Berthelot assay, which sensitively measures ammonia in both pure solutions and cell lysates. This method utilizes the Berthelot reaction to convert ammonia into indophenol, which is directly detected by LC-MS. In samples that have complex matrices, such as cell lysates (as shown in Figs 3 and 4), the MS Berthelot assay generates a distinct molecule, indophenol, which can only be derived from ammonia. Thus, indophenol provides a direct readout of ammonia levels. Therefore, the MS Berthelot assay significantly advances current methods for ammonia detection by filtering out the interfering molecules that often complicate analysis of ammonia in complex matrices such as soil, blood, seawater, and cell lysates. We speculate that the MS-Berthelot assay can be applied to these systems for a more sensitive and direct method to measure ammonia compared to techniques that are currently available.

The MS Berthelot assay has several advantages over current ammonia detection strategies, including specificity, sensitivity, and the ability to detect isotopologues. The colorimetric Berthelot reaction is complicated by interfering background reactions that absorb in the same spectrum as indophenol. In the MS Berthelot assay, the triple quadruple mass spectrometer filters out background reactions by selectively measuring ion counts of the mass:charge ratio of indophenol. Therefore, the MS-Berthelot assay resolves one of the major hurdles of ammonia measurement by eliminating the interference of background reactants in the readout. Furthermore, the colorimetric Berthelot assay for ammonia measurement has a lower limit of detection of ~7 μM, whereas the MS-Berthelot assay measures ammonia as low as 500 nM, increasing the sensitivity for ammonia detection 14-fold^11^. This sensitivity is not only an improvement on the colorimetric Berthelot assay, but also an improvement on commercial assays, which often detect a lower limit of ~10 μM ammonia.

In this study, the MS-Berthelot assay was performed on cell lysates that were isolated with a polar metabolite extraction, showing the potential for this assay to be multiplexed with metabolite profiling studies. For this type of analysis, the Berthelot Reaction must be performed on an aliquot of the initial metabolite isolation, and metabolite profiling be performed on the remaining unreacted portion, since the highly oxidizing conditions of the Berthelot reaction will alter the contents of the metabolite isolate (Fig. 3A). The MS-Berthelot assay requires only 2% of the initial metabolite isolation, and is therefore feasible to do in parallel with steady-state metabolomics experiments without negatively impacting metabolite detection.

Finally, the MS Berthelot assay was used to distinctly measure ammonia isotopologues (^14^NH_3_ and ^15^NH_3_). The MS Berthelot assay accurately measures ratios of ammonia isotopologues by analyzing ^14^N -indophenol (m/z:198) to represent ^14^NH_3_ and ^15^N-indophenol (m/z: 199) to represent ^15^NH_3_. Importantly, this analysis was performed on cellular lysates isolated by methanol extraction, and therefore, measurement of ammonia isotopologues can be integrated with nitrogen metabolic tracing studies. In this study, we provide one example of this in which the stable isotopologue of glutamine (^15^N-(amide)-glutamine) was used. We showed that using the MS Berthelot assay, cells with high glutaminase expression generate more ammonia from glutaminolysis than cells with low glutaminase expression. We detected this in both cell lysates and the media. In addition to glutamine, this technique can be applied to wide-range of metabolic tracing experiments involving other nitrogen-containing metabolites such as branched chain amino acids and nucleotides. Beyond mammals, the MS Berthelot assay is applicable to all organisms, as ammonia is a ubiquitious by-product of metabolism, and will significantly advance mechanistic studies of ammonia metabolism.

## Materials and Methods

### Mass Spectrometry

Samples were analyzed on a reverse phase ion-pairing chromatography coupled to tandem mass spectrometry (Agilent LC-MS). Analytes were eluted in buffer A (97% H_2_O, 3% MeOH, 10 mM Tributylamine, 15 mM Glacial Acetic Acid, pH 5.5) and buffer B (10 mM Tributylamine, 15 mM Glacial Acetic Acid in 100% MeOH). Samples were run on a ZORBAX Extend-C18, 2.1 × 150 mm, 1.8 μm (Agilent) starting with a flow rate of 0.25 mL/min for 2.5 minutes of buffer A, followed by a linear gradient (100% buffer A to 80% buffer A) for 5 minutes, followed by a linear gradient (80% buffer A to 55% buffer A) for 5.5 minutes, followed by a linear gradient (55% buffer A to 1% buffer A) for 7 minutes, followed by 4 minutes with (1% buffer A). Following each run, an acetonitrile backwash was utilized to clean the column followed by a 8 minute re-equilibration period of 100% buffer A. Samples were ionized (with negative polarity) using Agilent Jet Spray ionization; nebulizer 45 psi, capillary –2000 V, nozzle voltage: 500 V, sheath gas temperature 325 °C, and sheath gas flow 12 L/min. An Agilent 6470 Triple Quadrupole mass spectrometer was used for mass detection with a targeted method for indophenol. Using a 1 *u*g/mL indophenol standard (Sigma), the method was developed with a fragmentor voltage of 80 V, collision energy of 30 V, parent ion 198.1 and fragment 168.9. For ^15^N-indophenol, fragmentor voltage of 80 V, collision energy of 30 V, parent ion 199.1 and fragment 169.9 were utilized. Peaks were integrated in Mass Hunter (Agilent).

### Cell Culture

All cell lines were cultured in DMEM (Life Technologies) supplemented with 10% FBS (Life Technologies) and 1% penicillin and streptomycin (Invitrogen). For tracing studies using isotopic glutamine, glutamine-free RPMI (Life Technologies) supplemented with 2.0 mM ^15^N-(amide)-glutamine (Sigma), 10% FBS and 1% penicillin and streptomycin was used.

### RNA Isolation and RT-PCR

RNA was extracted using Direct-zol RNA Miniprep Kit (Genesee Scientific), and cDNA was synthesized using the iScript cDNA synthesis kit (Bio-Rad). Real-time qPCR was performed on a Light Cycler 480 (Roche) using PerfeCTa SYBR Green Fast Mix (Quanta BioSciences). Primers: RPLP0 (F): acgggtacaaacgagtcctg, RPLP0 (R): cgactcttccttggcttcaa, GLS (F): tgtcacgatcttgtttctctgtg, GLS (R): tcatagtccaatggtccaaag.

### Polar metabolite isolation

Metabolites were isolated directly from adherent cells. Media was aspirated and wells were quickly washed with 1 mL ice-cold PBS. Then, 1 mL of 80% HPLC-grade Methanol (BT Baker) for a 10 cm dish or 500 *u*L of ice-cold 80% HPLC-grade Methanol (BT Baker) for a 6-well dish were used to extract polar metabolites. Tissue culture dishes were incubated at −80 for 10 minutes, and scraped on dry ice. Lysates were centrifuged at 10,000 x g for 10 minutes and supernatants were either used for assays or stored at −80.

### Metabolic tracing

Lung cancer cell lines were incubated with 2.0 mM ^15^N-(amide)-glutamine for 24 hours. Metabolites were isolated from the media in a 10:1 80% MeOH: lysate ratio and from cells as previously described. The Berthelot reaction was performed on isolates and analyzed by LC-MS.

### The MS Berthelot assay

The Berthelot reaction was performed on pure ammonia standards made in 80:20 methanol:water or cellular lysates generated using the previously described method for polar metabolite extraction. Reactions in 80% MeOH were performed with a volumetric ratio of 10:1 (reactant mixture: sample). For example, 20*u*L of sample was reacted with 100*u*L Solution #1 and 100 *u*L Solution #2. In a cell lysate, the volumetric ratio used was 20:1 (reactant mixture: sample), in which 20 *u*L of sample would be reacted with 200 *u*L Solution #1 and 200 *u*L Solution #2. Solution #1 is composed of 100 mM Phenol (Sigma) and 50 mg/L sodium nitroprusside (Sigma). Solution #2 is composed of 0.38 M Dibasic Sodium Phosphate (Sigma), 125 mM NaOH, 1% sodium hypochlorite, available chlorine 10–15% (Sigma). Upon addition of the two reactant solutions, the samples are mixed and incubated at 37 °C for 40 minutes. All solutions and reactions should be stored at 4 °C until use for LC-MS analysis.

### Ammonia Measurement

Ammonia was measured using the Berthelot method as described above or using a commercial colorimetric assay (Abcam #ab83360).

### Statistical Analysis

Two-tailed student’s t-test was used to compare the means among experimental subgroups. All statistical tests had an alpha of 0.05 as the significance threshold. *P < 0.05, **P < 0.01, ***P < 0.005, ****P < 0.001, *****P < 0.0001.
